# Access to Harm Reduction Treatment Among Formerly Incarcerated Individuals During the COVID-19 Era

**DOI:** 10.1089/hs.2021.0037

**Published:** 2021-06-17

**Authors:** Tawandra L. Rowell-Cunsolo, Meghan Bellerose, Carl Hart

**Affiliations:** Tawandra L. Rowell-Cunsolo, PhD, is an Assistant Professor, Sandra Rosenbaum School of Social Work, University of Wisconsin-Madison, Madison, Wisconsin. Meghan Bellerose, MPH, is a Graduate Student, Mailman School of Public Health; and Carl Hart, PhD, is a Professor, Department of Psychology; both at Columbia University, New York, NY.

**Keywords:** COVID-19, Harm reduction, Medication for opioid use disorder, People who use drugs, Substance use disorder, Epidemic management/response

IN 2020, 1.8 million individuals were incarcerated in the United States.^1,2^ A disproportionate number of incarcerated individuals belong to racial and ethnic groups that made up 70% of the correctional population in 2019,^3^ despite accounting for only 40% of the US population. In particular, a substantial proportion of individuals released from incarceration each year are Black men who were convicted for drug-related offenses.^2-5^ This disproportionate representation of Black men in correctional settings is a product of systemic racism and structural disregard for Black life, both within and beyond the criminal legal system.^6^

All evidence suggests that substance use disorders are highly prevalent among incarcerated Americans; however, estimates of the scope of this problem vary. The most recent national estimates from the 2008–2009 National Inmate Survey indicate that up to 65% of incarcerated Americans met the criteria for a substance use disorder and an additional 20% were under the influence of drugs and alcohol at the time of their arrest.^7,8^ Despite the high rates of substance use disorders among this population, only 11% receive an adequate level of substance use treatment during incarceration.^9^ Postincarceration, substance use increases considerably,^10-12^ resulting in higher risks of reincarceration,^13^ contracting or transmitting infectious diseases such as HIV and hepatitis C,^10,14^ and overdose.^12,15,16^ The first year after release from incarceration is particularly challenging, as formerly incarcerated individuals attempt to rebuild their lives and maintain sobriety. Our previous research^17^ suggests that of those with past year criminal justice involvement (arrest, probation, or parole), Black Americans are less likely to have received substance use treatment.

Harm reduction strategies play a critical role in reducing drug-related overdoses, and yet as late as 2018, they were still generally unavailable in US jails and prisons^18^ and difficult to access following release.^19^ Harm reduction strategies, including medications for opioid use disorder, syringe exchange, naloxone distribution, and safe injection sites have proven to substantially reduce the likelihood of experiencing overdose and improve substance use treatment retention.^20^ Access to harm reduction services is essential for formerly incarcerated people who use drugs as they face a number of barriers to substance use treatment access, including lack of health insurance, limited motivation or readiness to change, long waiting lists to enter treatment, and transportation challenges.^21,22^

During the COVID-19 pandemic, access to substance use treatment has been further disrupted, as many programs have reduced their client sizes and eliminated in-person services to avoid transmission among clients and staff.^23^ As the initial surges of COVID-19 infection were concentrated in urban, high-density areas, which primarily house marginalized and minority individuals, these communities were especially vulnerable to experiencing service disruption.^24,25^ As a result, there is a pressing need to reduce barriers to harm reduction services in the United States. In this commentary we describe the challenges that formerly incarcerated people who use drugs experience as they return to their home communities, especially in the New York City area, and we discuss how these challenges are framed by systemic racism. We also offer recommendations to promote treatment engagement among this population, with an eye toward improving opportunities to access substance use disorder treatment and support postincarceration.

## Incarceration Treatment Gaps

During incarceration, access to substance use treatment and harm reduction services is limited. As of 2017, only Rhode Island had incorporated corrections-based harm reduction approaches into their statewide overdose prevention strategy.^26^ An analysis of the Rhode Island Department of Corrections model of screening and treatment with medications for opioid use disorder found that it contributed to a 60.5% reduction in postincarceration overdose deaths.^27^ This promising result and strong advocacy on the benefits of harm reduction approaches has increased efforts to incorporate harm reduction into state correctional settings; however, uptake has been slow.^28^ Additionally, in November 2020, the American Society of Addiction Medicine reported that only 4.6% of individuals referred to treatment for an opioid use disorder from the criminal justice system were currently receiving medications for opioid use disorder.^28^ Rapid decarceration during the COVID-19 pandemic has left this undertreated population of people who use substances with even fewer resources for postincarceration substance use disorder treatment support.

In the initial months of the COVID-19 pandemic, US jails and prisons released large numbers of inmates to reduce facility congestion and stem the spread of COVID-19. Between March and May 2020, 24 states including New York reported COVID-19-related releases, with 80 to 1,600 inmates released per state.^29^ Because infection rates are 3 times higher in correctional settings than in the general population, and incarcerated people of color are infected at disproportionate rates,^30,31^ these efforts were essential to reducing the spread of COVID-19 in marginalized populations. Although decarceration is a worthwhile goal, recently released people who use drugs have faced challenging transitions back to communities. With this surge of unexpected releases, reentry services have been stretched thin or eliminated.^32^ When implemented well, reentry services can reduce recidivism and mortality by offering assistance to recently released individuals based on their individual substance use treatment needs, social support, and employment goals.^32^ Without these services, recently released individuals have more trouble establishing community ties, gaining financial security, and resisting drug use or criminal activity.

## Postincarceration Treatment Gaps

Between March and December 2020, we conducted pilot phone surveys with 22 formerly incarcerated individuals who use substances that were returning to communities in the New York City area. The survey comprised both open- and closed-ended questions on postincarceration substance use and experience with substance use disorder treatment and support, and included the short-form American Society of Addiction Medicine Patient Placement Criteria.^33^ To recruit participants, we distributed recruitment flyers to community-based agencies and harm reduction sites throughout New York City that continued to provide services during the COVID-19 pandemic. English-speaking adults who had been released from incarceration within the past 3 years and reported using an illicit substance within the past 90 days were eligible to participate. Interested individuals called a number listed on the flyers to complete an eligibility screener, oral consent process, and a 45-minute phone survey. Those who completed the full survey received a $50 gift card by mail. The survey data were analyzed using IBM SPSS Statistics for Windows version 27 (IBM Corp, Armonk, NY). We received approval for this pilot study from the Columbia University Irving Medical Center Institutional Review Board (IRB-AAAS7578).

Among our participants, 16 identified as male, 5 as female, and 1 as transgender. Participants identified as non-Hispanic Black or African American (40.9%), followed by Hispanic or Latino (31.8%) and non-Hispanic White (22.7%). The majority were heterosexual (86.4%), single (63.6%), and had at least a high school diploma or GED (86.3%). Half of the participants (50.0%) had been released within the past 3 months.

Participants described transitioning to their communities as difficult and uncertain. In response to stressors associated with the pandemic, some reported returning to substance use in larger quantities after a period of abstaining. Our initial observations suggest that these stressors include housing instability, including uncertainty about how long they could remain in temporary hotel accommodations, the need to live with substance-using friends or family, grim prospects for future employment, and trouble establishing a social network or daily routine that helps them abstain from substance misuse. These preliminary findings are aligned with emerging research indicating that people who use drugs face a number of unique consequences postincarceration, including navigating complex institutional barriers to accessing social benefits and substance use disorder treatment.^34^

### Housing

In New York City, formerly incarcerated individuals with a history of substance use are typically faced with limited housing choices once they are released from incarceration. According to a Coalition for the Homeless report, 54% of all individuals released from prison in New York City entered the shelter system directly in 2017, up from 23% in 2014.^35^ Postrelease housing is a particular concern for people of color, as racism embedded within historical policies and contemporary practices have led them to occupy a higher percentage of substandard and temporary housing.^36^ Today, formerly incarcerated Black men have far higher rates of homelessness than White or Hispanic men (124 vs 81 and 82, respectively, per 10,000 formerly incarcerated people).^37^

The COVID-19 pandemic has exacerbated these housing challenges and disparities. Our previous research on formerly incarcerated people who use drugs in New York City suggests that they are generally concentrated in congregate settings, including homeless shelters and single-resident occupancy hotels,^38^ which have been the source of COVID-19 outbreaks, accelerating the spread of COVID-19.^39,40^ Some of these environments house hundreds of residents who share living spaces and equipment, which makes it difficult to adhere to guidelines from the Centers for Disease Control and Prevention urging social distancing, avoidance of crowds, and self-isolation to reduce exposure. Indeed, housing was a primary concern for the majority of our participants. Over a third (36.4%) considered themselves homeless, including 6 participants living in shelters and 4 temporarily housed in hotels during the COVID-19 pandemic.

### Social Support

In addition, an often overlooked yet critical component to addressing substance use disorders and the recovery process is the support from one's social network. For recently incarcerated people who use drugs, support from friends and families can be one of the most helpful resources to maintaining sobriety.^41,42^ Although many formerly incarcerated people who use drugs have exhausted the delicate resources of their family and friends, often due to substance use-associated problems (eg, fights over money, stealing),^12^ the social interaction with those who can provide encouragement is important for a number of reasons. First, friends or family members of people who use substances may be able to identify when a return to or increase in drug use is imminent, enabling them to provide emotional support to help strengthen the resolve to abstain from using drugs.^43^ Second, research also suggests that communication and support from family members and friends promote treatment entry and engagement.^44^ Establishing a recovery support network comprised of others in recovery could also promote attachment to a community. Formerly incarcerated individuals experience a number of stressors as they return to communities, and just having someone to commiserate with may help them cope and prevent relapse.^42^

Many people who use drugs are incarcerated for offenses associated with their substance use,^7^ then separated from their loved ones with limited options for meaningful interactions. This scenario has especially been difficult for people of color who typically spend more time incarcerated for drug-related offenses, even though their substance use is comparable to those of White individuals.^45^ The impact of harsh penalties on Black people who use drugs has resulted in the consistent removal of young men from communities for prolonged periods of time, which has had severe economic and health-related consequences for this population.^46^ These harsh penalties have effectively impacted the maintenance of healthier stable relationships and social networks, an essential element of sustained substance use disorder recovery.^12^

Our pilot study findings revealed that adherence to COVID-19 social distancing guidelines has strained family and romantic relationships, complicating efforts to establish stable, health-promoting ties for people who use drugs and are being released from correctional institutions during the pandemic. In particular, participants who were temporarily housed in hotels following release reported difficulty establishing a social network or accessing services in unfamiliar neighborhoods far from the city center.

## Improving Harm Reduction Access During the COVID-19 Pandemic

Fortunately, some obstacles to postincarceration harm reduction services are temporarily being removed. For instance, the US federal government has waived initial in-person assessments for initiating buprenorphine and increased flexibility for dispensing take-home methadone.^47^ This decision is crucial to providing people who use drugs and are moving from custody to communities with the necessary substance use disorder support and resources to prevent negative consequences associated with substance misuse. Individual states have also moved to reduce service disruptions during the COVID-19 pandemic and to ensure access to harm reduction services.^48^ In March 2020, Maine Governor Janet Mills issued an executive order on suspending the existing 1-to-1 syringe exchange rule so that people who use drugs could take home multiple syringes, and the Oregon Housing Authority authorized syringe service programs to provide curbside no-contact pickups. In Pennsylvania, the Secretary of Health permitted community organizations to distribute naloxone through the mail, and in New York, 22 of the 23 formal syringe service programs began peer-delivery services.^48^ Such flexibility and innovation may be needed in the future as there may be additional service-related disruptions should another wave of COVID-19 outbreaks emerge.

Individual agencies have also spearheaded efforts to ensure that harm reduction services are available during the pandemic. During the COVID-19 pandemic, a greater number of formerly incarcerated individuals may be choosing to use drugs alone in order to maintain social distancing guidelines or due to disruptions in their drug use networks, thereby increasing their risk of accidental overdose.^49^ The organization Never Use Alone is mitigating this risk by offering virtual injection supervision by phone during the pandemic.^50^ While these innovations have surely had an impact, more systemic changes are needed.

## Recommendations

Based on the potential challenges to accessing harm reduction services and substance use disorder treatment raised in this article, we would like to offer a few recommendations that may be useful in mitigating the consequences associated with the pandemic's impact on formerly incarcerated people who use substances, particularly people of color.

Our first recommendation is to provide a range of alternative housing options. New York City is currently using hotels and other temporary housing solutions to address housing-related shortages and an overreliance on congregate housing. However, this is a temporary solution that may result in anxiety and additional negative consequences when residents are eventually required to find new places to live. In addition, during the time that recently released people reside in temporary hotel placements, which are often located far from the city center or in expensive tourist areas, they are unable to begin forming community ties and social support systems, which may lead to increased likelihood of recidivism.^51^ Relapse and recidivism rates are highest among those who are unstably housed.^52^ More permanent housing solutions should be considered to promote stability and healthy reintegration among formerly incarcerated people who use drugs. This is particularly important for people of color who tend to reside in underresourced communities that lack the infrastructure to offer high-quality recovery services.^53,54^

The second recommendation is to promote ongoing access to healthcare services by making permanent the federal policy eliminating mandatory in-person visits for opioid treatment services and support, which was implemented in response to the initial waves of the COVID-19 pandemic. In particular, using phone- and internet-based health services to provide screening and deliver treatment and related support may be useful in addressing significant gaps in accessing healthcare services among this population. It is important to consider equity issues that may arise if some do not have access to reliable internet or mobile phone services; however, adoption of mobile technology among criminal justice-involved populations is reportedly high, in some instances above 80%.^55^ Virtual care visits for substance use disorder treatment can be more easily integrated into other programs or aspects of correctional and clinical care,^56^ which may result in greater treatment compliance for other conditions as well.

Finally, pharmacists should have a more active role in substance use disorder treatment delivery. Over 300,000 pharmacists in the United States are trained to provide counseling on medications and detect adverse events and also receive training on harm reduction and addiction medicine.^57^ Their skills and training have been underused during the pandemic. In some countries, registered pharmacies are able to dispense methadone and buprenorphine.^58^ In the United States, in contrast, only a fraction of licensed physicians (36,715 out of 926,119 in 2016) are waivered to prescribe buprenorphine, of which the majority have a 30 patient limit.^19^ Strong collaborations between physicians and pharmacists should be established to administer treatment, resolve prescribing errors, and share specialized knowledge; these strategies may help reduce reliance on providers who may be overburdened during the pandemic.^59^ These collaborations may also reduce racial disparities in access to medications for opioid use disorder beyond the pandemic. In 2019, buprenorphine use in New York City and nationally is concentrated in higher-income neighborhoods with a low percentage of people of color.^60,61^ Therefore, collaborations designed to improve the efficiency of prescribing buprenorphine, in particular, should be explored to address deficiencies in the system.^62^ Collaborative prescribing environments in New York City are particularly important since opioid-related overdose rates have almost doubled, and are steadily increasing among people of color and residents in high-poverty communities.^63^

## Conclusion

In this commentary, we demonstrate that recently incarcerated people who use drugs are returning to communities without the support structures in place that are needed to reduce or abstain from substance use. Most of the challenges we have identified among this population, including unstable housing, fragile social support systems, and limited access to substance use disorder treatment, are not new; however, they have been accentuated by decarceration and social distancing measures during the COVID-19 pandemic. Given the harsh impact of COVID-19 on marginalized and minority communities, we hope that expanding housing options and improving access to medications for opioid use disorder will help address barriers to harm reduction services and substance use disorder support for formerly incarcerated people who use drugs, a medically underserved segment of the population.
